# Fungal diversity regulates plant-soil feedbacks in temperate grassland

**DOI:** 10.1126/sciadv.aau4578

**Published:** 2018-11-28

**Authors:** Marina Semchenko, Jonathan W. Leff, Yudi M. Lozano, Sirgi Saar, John Davison, Anna Wilkinson, Benjamin G. Jackson, William J. Pritchard, Jonathan R. De Long, Simon Oakley, Kelly E. Mason, Nicholas J. Ostle, Elizabeth M. Baggs, David Johnson, Noah Fierer, Richard D. Bardgett

**Affiliations:** 1 School of Earth and Environmental Sciences, Michael Smith Building, The University of Manchester, Oxford Road, Manchester M13 9PT, UK.; 2 Cooperative Institute for Research in Environmental Sciences, University of Colorado, Boulder, CO 80309, USA.; 3 Department of Ecology and Evolutionary Biology, University of Colorado, Boulder, CO 80309, USA.; 4 Freie Universität Berlin, Institut für Biologie, Plant Ecology, D-14195 Berlin, Germany.; 5 Berlin-Brandenburg Institute of Advanced Biodiversity Research (BBIB), D-14195 Berlin, Germany.; 6 Institute of Ecology and Earth Sciences, The University of Tartu, Lai 40, 51005 Tartu, Estonia.; 7 The Global Academy of Agriculture and Food Security, The Royal (Dick) School of Veterinary Studies, The University of Edinburgh, Midlothian EH25 9RG, UK.; 8 Department of Terrestrial Ecology, Netherlands Institute of Ecology, P.O. Box 50, 6700 AB Wageningen, Netherlands.; 9 Centre for Ecology & Hydrology, Lancaster Environment Centre, Library Avenue, Bailrigg, Lancaster LA1 4AP, UK.; 10 Lancaster Environment Centre, Lancaster University, Lancaster LA1 4YQ, UK.

## Abstract

Feedbacks between plants and soil microbial communities play an important role in vegetation dynamics, but the underlying mechanisms remain unresolved. Here, we show that the diversity of putative pathogenic, mycorrhizal, and saprotrophic fungi is a primary regulator of plant-soil feedbacks across a broad range of temperate grassland plant species. We show that plant species with resource-acquisitive traits, such as high shoot nitrogen concentrations and thin roots, attract diverse communities of putative fungal pathogens and specialist saprotrophs, and a lower diversity of mycorrhizal fungi, resulting in strong plant growth suppression on soil occupied by the same species. Moreover, soil properties modulate feedbacks with fertile soils, promoting antagonistic relationships between soil fungi and plants. This study advances our capacity to predict plant-soil feedbacks and vegetation dynamics by revealing fundamental links between soil properties, plant resource acquisition strategies, and the diversity of fungal guilds in soil.

## Teaser

Plant growth dynamics are shaped by the diversity of associating pathogenic, saprotrophic, and mutualistic soil fungi.

## INTRODUCTION

The accumulation of host-specific pathogenic fungi in plant rhizospheres has been identified as an important driver of negative density dependence in plant populations. These negative plant-soil feedbacks underlie plant species coexistence and positive diversity-productivity relationships (*1*–*6*). The role of soil pathogens in regulating forest and grassland communities has been deduced primarily from seedling mortality and plant tissue damage combined with soil inoculation and fungicide treatments (*3*–*5*, *7*, *8*). Hence, while many studies have demonstrated the negative effect of soil biota on plants, the identity, specificity, and diversity of fungal pathogens in natural soils remain largely unknown (*9*–*11*). Moreover, host-specific changes in the abundance and composition of saprotrophic and mycorrhizal fungi also contribute to both negative and positive feedbacks to plant performance, but their relative importance remains poorly understood (*7*, *10*, *12*, *13*).

Pathogenic, mutualistic, and saprotrophic fungi can drive plant-soil feedbacks in complex ways. The net outcome for plant growth will depend on antagonistic and synergistic interactions within the hyperdiverse soil microbiome (*10*, *14*). The nature of interactions between plants and soil biota is likely to be modified by plant functional traits that reflect the trade-off between plant resource acquisition and conservation via improved natural enemy defence and longevity [known as growth-defence trade-off and leaf economics spectrum (*12*, *15*, *16*)]. Moreover, soil abiotic factors including pH and nutrient availability can have strong effects on the composition of soil microbial communities (*17*–*19*) and plant functional traits (*20*), with soil edaphic factors often varying considerably even across small spatial scales (*21*). While it is well established that small-scale variation in soil abiotic properties can be important in determining plant competitive interactions and diversity (*21*, *22*), the role of soil properties in mediating the context dependency of plant-soil feedbacks has largely been overlooked. In addition to effects mediated by changes in microbial composition, plant and soil properties may modify plant-soil feedbacks via changes in microbial diversity, which has recently been demonstrated to enhance multiple ecosystem functions including nutrient cycling and productivity (*23*, *24*). Here, we tested (i) how the composition and diversity of putative pathogenic, mutualistic, and saprotrophic fungi, and the composition of associated bacterial and protist communities, control the outcome of plant-soil feedbacks and (ii) whether plant traits and variation in soil abiotic properties modulate plant-fungal interactions in temperate grassland.

## RESULTS AND DISCUSSION

**Fig. 1:**
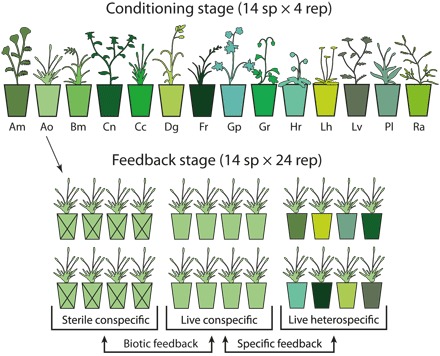
Experimental design. In the conditioning stage, monocultures of 14 common grassland species, each replicated four times, were grown for three growing seasons in large mesocosms filled with soil collected from a single grassland. In the feedback stage, eight newly germinated seedlings of each plant species were transplanted as single individuals back into (i) four replicate conspecific soils (i.e., previously occupied by the same species), (ii) four replicate conspecific soils sterilized with gamma irradiation, and (iii) eight randomly selected heterospecific soils (i.e., previously occupied by other species). Plants were harvested after 60 days of growth. Biotic feedback was calculated as the difference between mean log_e_-transformed dry mass of two plants grown in each live conspecific replicate soil and two plants grown in the corresponding sterilized conspecific replicate soil (resulting in a total of four feedback values per plant species). Specific soil feedback was calculated as all pairwise differences between log_e_-transformed dry mass of plants in each conspecific live soil and in each soil conditioned by other species (64 feedback values per plant species). Am, *Achillea millefolium*; Ao, *Anthoxanthum odoratum*; Bm, *Briza media*; Cc, *Cynosurus cristatus*; Cn, *Centaurea nigra*; Dg, *Dactylis glomerata*; Fr, *Festuca rubra*; Gp, *Geranium pratense*; Gr, *Geum rivale*; Hr, *Hypochaeris radicata*; Lh, *Leontodon hispidus*; Lv, *Leucanthemum vulgare*; Pl, *Plantago lanceolata*; Ra, *Rumex acetosa*.

Replicated monocultures of 14 common temperate grassland species, encompassing a broad spectrum of functional traits (*25*), were established in field-based mesocosms using soil collected from a mesotrophic grassland in northern England, United Kingdom (Fig. 1). After three growing seasons, plant species caused distinct changes in soil abiotic properties (fig. S1), especially nitrate availability (adjusted *R*^2^ = 0.63, *F*_13,41_ = 7.9, *P* < 0.001). Physicochemical properties, such as cation exchange capacity, pH, and soil carbon (C), nitrogen (N), and phosphorus (P) concentrations, formed another axis of variation reflecting inherent soil fertility that was not significantly affected by conditioning with different plant species (adjusted *R*^2^ = −0.06, *F*_13,41_ = 0.75, *P* = 0.706; fig. S1). Therefore, the experiment captured variation in soil abiotic properties resulting from both plant species conditioning and inherent variability in soil fertility at the site of soil collection. This enabled us to gauge the relative importance of both biotic and abiotic drivers of plant-soil feedbacks.

**Fig. 2:**
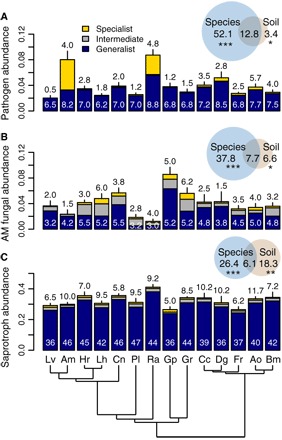
The relative abundance of fungal sequences belonging to different fungal guilds. (**A**) Putative plant pathogenic fungi. (**B**) AM fungi. (**C**) Saprotrophic fungi. Means + SE are shown (*n* = 55). Plant species identity (blue circles) primarily explained variance in the relative abundances of fungal pathogens and AM fungi, while both plant identity and variation in soil physicochemical properties (brown circles) explained differences in saprotroph abundances. Variance explained is based on adjusted *R*^2^. The numbers above the bars show the mean richness of specialist taxa (those that occur in fewer than 5 plant species), and the numbers in white show the mean richness of generalist taxa (those that occur in more than 10 plant species). Plant phylogeny is shown at the bottom of the graph. See Fig. 1 for species abbreviations. *P < 0.05, **P < 0.01, and ***P < 0.001.

We characterized soil microbial communities using high-throughput marker gene sequencing and found that the composition of soil fungal and protist communities varied significantly between soils conditioned by different plant species (fig. S1). Plant species identity had little influence on soil bacterial communities, which were primarily determined by soil abiotic conditions (fig. S1). The distinct effects of plant species on soil fungal communities became more pronounced when fungi were split into putative plant pathogens and saprotrophs [identified by comparison of taxonomy to the public database FUNGuild (*26*)] and arbuscular mycorrhizal (AM) fungi (Fig. 2 and fig. S1). The relative abundance of the three fungal guilds differed significantly between soils conditioned by different species. Plants left the strongest legacy on the relative abundance of putative pathogens and AM fungi, while saprotrophs were more affected by soil abiotic conditions (Fig. 2). Plant species also varied widely in their tendency to accumulate specialist pathogenic fungi (richness of specialist putative pathogens: adjusted *R*^2^ = 0.32, *F*_13,41_ = 2.9, *P* = 0.004) but not in the accumulation of specialist AM and saprotrophic fungi (richness of specialist AM fungi: adjusted *R*^2^ = 0.19, *F*_13,41_ = 2.0, *P* = 0.050; richness of specialist saprotrophic fungi: adjusted *R*^2^ = −0.05, *F*_13,41_ = 0.82, *P* = 0.640; Fig. 2 and figs. S2 and S3). Therefore, plant species left distinct soil legacies on the relative abundances and diversity of different fungal groups, with these changes expected to elicit specific feedbacks to subsequent plant growth.

For the feedback phase of the study, seedlings of each plant species were planted as single individuals into replicate soils that were previously conditioned by conspecifics and eight randomly selected heterospecific soils, each conditioned by a different plant species (Fig. 1). A second set of seedlings was planted in conspecific soil that had been sterilized via gamma irradiation. Two plant-soil feedback indices were calculated (Fig. 1). Biotic feedback was calculated as the ratio of plant biomass when grown in live conspecific soil versus the same soil sterilized by gamma irradiation, which reflects the net outcome of plant interactions with soil biota. Specific feedback was calculated as the ratio of plant mass when grown in live conspecific soil versus live heterospecific soil, and therefore describes responses to specific changes in soil properties caused by different plant species. More negative values of this index are indicative of poorer plant growth on soil previously occupied by the same species compared to another species, and this process is predicted to promote stable species coexistence in plant communities (*6*, *7*).

**Fig. 3:**
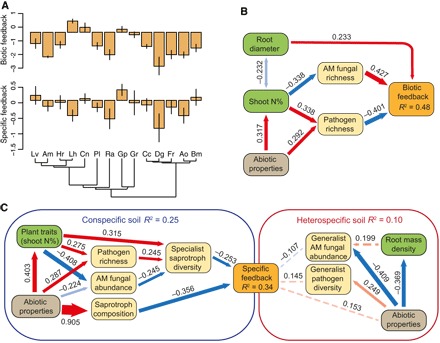
Effects of plant species identity, plant traits, and soil properties on plant-soil feedbacks. (**A**) Variation in biotic and specific feedbacks (means ± SE) among studied plant species. Negative values indicate greater plant growth in sterilized soil (top) or soil conditioned by heterospecifics (bottom). See Fig. 1 for species abbreviations, fig. S4 for raw data, and Materials and methods for details on feedback calculation. (**B**) Path analysis of variables influencing biotic feedbacks. (**C**) Path analysis of variables influencing specific feedbacks. Red arrows indicate positive relationship, and blue arrows indicate negative relationship. Solid colors indicate significant relationships (*P* < 0.05), and semitransparent arrows indicate marginally nonsignificant relationships (*P* < 0.1). For heterospecific soil properties, nonsignificant relationships (0.1 < *P* < 0.4) are shown with dashed lines. Standardized path coefficients are shown. All tests are based on *n* = 55. Abiotic properties and plant traits refer to the first principal components of soil physicochemical properties (more positive values represent soil with higher fertility) and of plant trait data (more positive values represent more resource-acquisitive traits), respectively; saprotroph composition refers to the first principal coordinate of saprotrophic fungal composition.

**Fig. 4:**
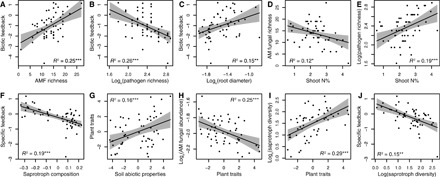
Relationships between plant traits, soil properties, and the strength of plant-soil feedbacks. (**A** to **J**) Biotic feedback refers to plant response to soil sterilization, and specific feedback refers to plant responses to conspecific versus heterospecific soils (as described in Fig. 1). Plant traits, soil abiotic properties, and saprotroph composition refer to the first principal components of plant trait data and soil abiotic properties and the first principal coordinate of fungal saprotroph communities, respectively. Plant trait axis correlates positively with shoot N% and specific leaf area and negatively with root dry mass density in soil and shoot carbon-to-nitrogen ratio (fig. S1). AM fungal abundance refers to the relative abundance of sequence reads classified as AM fungi. Saprotroph diversity refers to the exponential Shannon diversity of specialist saprotroph fungi. Solid lines indicate regression lines, and shaded areas indicate 95% confidence intervals. *P < 0.05, **P < 0.01, and ***P < 0.001.

We found that plant growth was, on average, 2.8 times greater in sterilized relative to nonsterilized conspecific soil (i.e., negative biotic feedback). Such an effect could be due to a release of nutrients from dead microbial biomass in sterilized soil. However, variation in biotic feedback was not explained by the amount of soluble nitrogen [i.e., the sum of nitrate, ammonium, and dissolved organic nitrogen (DON)] released into the soil (*R*^2^ = 0.04, *F*_1,53_ = 2.1, *P* = 0.154). The magnitude of negative biotic feedback varied across plant species (adjusted *R*^2^ = 0.66, *F*_13,41_ = 9.2, *P* < 0.001) but could not be predicted by phylogenetic relationships between species (Blomberg’s *K* = 0.22, *P* = 0.441; Pagel’s λ = 0.22, *P* = 0.389; Fig. 3A). Instead, the magnitude of biotic feedback was best predicted by the richness of putative fungal pathogens and AM fungi in the soil (*R*^2^ = 0.48; path analysis model fit χ^2^ = 16.5, *P* = 0.350, Comparative Fit Index (CFI) = 0.99, Root Mean Square Residual (SRMR) = 0.076; Fig. 3B). Biotic feedback was consistently more negative for those plant species that accumulated more diverse communities of putative fungal pathogens, but less negative for plant species that supported soils with greater relative abundance and richness of AM fungi (Fig. 4, A and B, and table S1). Despite pathogenic oomycete genera *Pythium* and *Phytophtora* comprising up to 20% of all protist sequences, higher abundances or richness of these pathogenic protist taxa were not associated with more negative plant-soil feedbacks (table S2).

The relative abundance of host-specific pathogenic fungi also contributed to biotic feedback (*R*^2^ = 0.10, *P* = 0.018), but the effect was considerably weaker than that of the richness of putative pathogens and AM fungi (*R*^2^ = 0.26, *P* < 0.001 and *R*^2^ = 0.25, *P* < 0.001, respectively; table S1). In support of this conclusion, variation in biotic feedback was more correlated with differences in the richness of putative pathogenic (Mantel test: rho = 0.21, *P* = 0.005) and AM fungi (rho = 0.21, *P* = 0.014) than with differences in the composition of these fungal groups (rho = −0.05, *P* = 0.695 and rho = 0.12, *P* = 0.050, respectively). These findings provide compelling evidence that plant-soil feedback is regulated by the diversity of soil fungi and indicate that diversity effects of soil organisms are not only positive (*23*, *24*) but also negative via interactions between plants and putative fungal pathogens. Our results also suggest that, in addition to the known positive effect of AM fungal presence on plant growth (*27*, *28*), less negative plant-soil feedbacks are likely to occur in soils with higher AM fungal richness.

Two plant traits, shoot N concentration and mean root diameter, were identified as significant predictors of the magnitude of biotic feedback. Plant species with finer roots suffered a more negative impact from soil biota (Fig. 4C), and species with higher shoot N concentrations accumulated fewer mycorrhizal fungi and attracted a more diverse community of putative fungal pathogens, resulting in a more negative net interaction with the soil microbiome (Figs. 3B and 4, D and E). Previous studies have shown that plant species with high growth rates, short life spans, and fine roots tend to suffer from more negative plant-soil feedbacks (*12*, *15*, *16*). Our results identify mechanistic links between plant resource acquisition strategy, putative pathogenic and mycorrhizal fungi, and the outcome for plant-soil feedbacks. Rather than a single factor determining the outcome of plant-soil feedbacks, plant traits and soil biotic and abiotic properties were closely interlinked and jointly determined the net effect of soil biota on plant growth (Fig. 3B and fig. S5). As plant traits affected the diversity of soil fungal pathogens and mutualists in opposing ways, the links between plant traits and soil microbial communities only became evident when considering different guilds of plant-associated fungi separately, but were not detectable when considering the composition of whole fungal, bacterial, and metazoan communities (*25*). However, functional roles of many soil microorganisms, especially bacteria, remain unknown; addressing this knowledge gap is critical for understanding how plants affect and respond to changes in soil microbiome.

Plants also accumulated, on average, 24% more biomass when grown on live soil conditioned by other species compared to soil previously occupied by conspecifics, indicating negative specific feedback between plants and soil communities. However, variation in specific feedback could not be explained by plant species identity (adjusted *R*^2^ < 0.01, *P* = 0.181 based on a randomization test; Fig. 3A). Contrary to previous studies on leaf pathogens demonstrating high levels of disease transmission among closely related species (*8*, *29*), the phylogenetic distance between plant species did not explain variation in specific feedback (Blomberg’s *K* = 0.17, *P* = 0.688; Pagel’s λ < 0.01, *P* = 1; Fig. 3A) or predict the proportion of putative pathogens, AM fungi, or saprotrophs shared between plant species (fig. S6). However, the nature of interactions between plants and soil fungi was significantly modified by variation in soil abiotic properties, with more negative specific feedbacks observed on inherently fertile soil (path analysis model fit χ^2^ = 20.1, *P* = 0.985, CFI = 1, SRMR = 0.059; Fig. 3C and table S1). The overall composition of bacterial, protist, and fungal communities in soil was strongly correlated with each other (table S2) and mediated the negative effect of soil fertility on plant growth in conspecific, but not heterospecific, soil (Figs. 3C and 4F). The significant relationship between specific feedback and the composition of saprotrophic fungi in conspecific soil (Fig. 4F) indicates that some saprotrophic fungi may shift to a biotrophic mode when continuously exposed to plant monocultures (*30*, *31*).

Soil abiotic properties also modified specific feedback indirectly via phenotypic plasticity in plant traits: More fertile soils shifted plant traits from conservative to more resource acquisitive (Fig. 4G). This effect was associated with an increase in the richness of putative pathogens, a decrease in the relative abundance of mycorrhizal fungi (Figs. 3C and 4H), and a higher diversity of specialist saprotroph fungi, which promoted more negative specific feedback to plant growth (Figs. 3C and 4, I and J). These effects could be due to AM fungi having a protective effect on plant tissues and, conversely, high pathogen diversity enhancing the abundance of dead tissue available to specialist saprotrophs (*32*–*34*). While exhibiting strong links with different fungal guilds on conspecific soils, plants showed little sensitivity to variation in microbial legacies left behind by heterospecifics (Fig. 3C and fig. S4). In support of this conclusion, we found that dissimilarities in pathogenic and mycorrhizal fungal communities, and phylogenetic distances between plant species, were poor predictors of plant growth responses to soils conditioned by different plant species (table S3).

Together, our findings identify novel mechanistic links between the outcome of plant-soil feedback for plant growth and the diversity of putative pathogenic, saprotrophic, and mycorrhizal fungi. Further, they indicate that the ability to predict plant-soil feedbacks requires better understanding of plant interactions with diverse communities of plant pathogens and mutualists, rather than single host-specific pathogens (*10*, *14*). The presence and absence of pathogenic and mutualistic fungi (*1*), and different groups of mycorrhizal fungi [ectomycorrhizal and AM fungi (*7*)], have been considered to be key regulators of plant-soil feedbacks. Our analysis demonstrates that the richness of these groups is also critical in determining plant performance, and thus provides a further route by which biodiversity can regulate community dynamics and ecosystem function. Previous studies have primarily focused on beneficial effects of soil biodiversity, and in particular of individual guilds such as AM fungi (*35*), on plant growth (*23*, *24*, *35*). Our results advance on these findings by showing that diversity effects of soil fungi on plant-soil feedback vary markedly between key guilds of soil fungi, and the collective outcome of these responses determines plant productivity and likely plant competitive interactions. Further, we show plant nutrient acquisition strategy and soil fertility to be important regulators of plant-soil feedback, with resource-exploitative plant species and fertile soils promoting negative feedbacks. Understanding relationships between plant resource acquisition strategies and the diversity of important soil fungal guilds should enhance our capacity to predict plant-soil feedbacks and vegetation dynamics in terrestrial ecosystems. Our study focused on temperate grassland species. However, traits such as leaf N concentration and root diameter, two of the key predictors of plant-soil feedback in our study, reflect variation in plant strategies that operate at the global scale (*36*, *37*). Empirical data from other ecosystems are needed to explore the generality of the relationships observed here, including the importance of fungal diversity for plant-soil feedbacks in the context of other abiotic and biotic factors.

## MATERIALS AND METHODS

### Soil conditioning stage

In the conditioning stage, monocultures of 14 common grassland species were grown for three growing seasons in large mesocosms (55 liters) filled with soil collected from a mesotrophic grassland in the Yorkshire Dales, United Kingdom (54°11′38.7″N, 2°20′54.4″W). The mesocosms were placed outdoors at the site of soil collection, and each monoculture was replicated four times. *A. odoratum* was represented by three mesocosms due to plant mortality in one of the mesocosms (hence, total *n* = 55). Soil collected from the grassland site exhibited natural spatial heterogeneity in soil properties, and this variation was preserved when filling the mesocosms with soil collected randomly from across the grassland. Plant traits, soil abiotic properties, and microbial communities were measured for each mesocosm at the end of the conditioning stage [data were described and partially used in (*25*)]. Briefly, leaves from at least three plants in each mesocosm were sampled at peak biomass in the third growing season and stored at 4°C before analysis. In addition, a soil core with a diameter of 6.8 cm was taken from each mesocosm and immediately sieved to 4 mm and subsampled for DNA extraction. Roots not passing through the sieve were washed free of soil before analysis. Leaf and root traits were quantified following standardized protocols (*38*). Briefly, leaf area, root length, and diameter were determined by scanning fresh samples and analysis with WinRHIZO (Regent Instruments Inc., Ville de Québec, Québec, Canada). The samples were weighed fresh and dried at 60°C for 48 hours, and specific leaf area, specific root length, and leaf and root dry matter content were calculated. Shoot and root N and C contents were measured on an Elementar Vario elemental analyzer (Hanau, Germany). The remaining sieved soil was used to quantify abiotic properties as in (*39*, *40*). Fresh soil samples were extracted with 1 M KCl and 0.5 M K_2_SO_4_ and analyzed for available NH_4_, NO_3_, and DON on a Seal AA3 Segmented Flow Multi-chemistry analyzer (Mequon, WI, USA) and for dissolved organic carbon on a Shimadzu 5000A total organic carbon analyzer (Asia Pacific, Kyoto, Japan). Dried ground subsamples were analyzed for soil C and N concentrations on an Elementar Vario EL elemental analyzer (Hanau, Germany), and soil P concentrations were determined by ignition (550°C, 1 hour) and extraction in 1 M H_2_SO_4_ for 16 hours, with phosphate detection by automated neutralization and molybdate colorimetry on a Lachat QuikChem 8500 autoanalyzer (Hach Ltd., Loveland, CO, USA). Exchangeable cations (Al, Ca, K, Mg, Mn, and Na) were extracted in 0.1 M BaCl_2_ for 2 hours (1:30 soil-to-solution ratio) and detected by inductively coupled plasma optical emission spectrometry on an Optima 7300 DV spectrometer (PerkinElmer Ltd., Shelton, CT, USA), with effective cation exchange capacity calculated as the sum of the positive charge of all exchanged cations. All plant traits and soil abiotic properties were checked for the assumption of normal distribution and were log_e_ transformed as necessary before use in the models described below.

### Feedback stage

In the feedback stage of the experiment, eight seedlings of each plant species were transplanted as single individuals back into (i) four conspecific soils (two replicate seedlings per soil), (ii) four conspecific soils sterilized with gamma irradiation (dose > 25 Gy, two replicate seedlings per soil), and (iii) eight heterospecific soils, each conditioned by a different, randomly selected species. Heterospecific soils were randomly assigned so that soil from each mesocosm was equally represented in the final dataset. Twenty-four seedlings per species and a total of 336 seedlings were initially planted (323 seedlings were measured at harvest). Seeds were germinated on sterile sand, and seedlings were transplanted into 0.5-liter pots filled with 300 g of soil (dry weight). All soils were left to settle in the pots for a week and were flushed with water three times to reduce the effects of sterilization on soil nutrient availability. Pot positions were randomized twice during the course of the experiment. Pots were watered daily, and soil moisture was brought to 60% water holding capacity at the start of the experiment and reset to this level twice during the course of the experiment. Plants were grown in a glasshouse with a temperature of 22°C and additional lighting with day/night cycle of 16/8 hours. Plant shoots and roots were harvested after 60 days of growth and dried at 70°C for 48 hours.

Two plant-soil feedback indices were calculated:

1) Biotic feedback was calculated as the difference between log_e_-transformed dry mass of plants in live and sterilized conspecific soil. The dry mass of two seedlings grown on soil from the same mesocosm was averaged before the calculation, resulting in four feedback values per plant species to reflect independent soil replicates.

2) Specific soil feedback was calculated as the difference between log_e_-transformed dry mass of plants in conspecific live soil and in soil conditioned by other species. Feedback was calculated for all pairwise combinations of plant dry mass in conspecific versus heterospecific soils within each plant species (i.e., 64 combinations per species). These pairwise feedback values were used in the subsequent data analyses, but statistical analyses were tailored to account for the use of the same plants in multiple feedback calculations and to obtain a conservative estimate of statistical significance for relationships between feedback and soil properties (there were 55 independent soil replicates). The significance of plant species identity in explaining variation in specific feedback was estimated by the comparison of the observed *F* statistic to the null distribution obtained using a restricted randomization approach (999 iterations; species identity was randomly shuffled between groups of feedback values derived from each independent soil replicate). Adjusted *R*^2^ was calculated with sample size corrected to 55 independent observations. Mean feedback values (and their SEs) for each plant species shown in Fig. 3A were derived as coefficients from linear models that were performed for each plant species separately and included log_e_-transformed dry mass as a response variable and treatment (two levels: conspecific and heterospecific as the reference level) as a fixed factor [as described in (*41*)].

### Microbial sequencing data

Microbial community structure was determined as in (*25*). Briefly, DNA was extracted from soil samples collected from each mesocosm at the end of the conditioning stage, and marker genes [the internal transcribed spacer of the ribosomal RNA (rRNA) operon for fungi, 16*S* rRNA for bacteria, and 18*S* rRNA for protists] were polymerase chain reaction amplified and used to characterize the microbial communities. The amplicons were sequenced on an Illumina MiSeq instrument with 2 × 151 base pair kits.

Exact sequence variant (ESV; also known as unique sequence variants and zero-radius operational taxonomic units) counts were determined from raw sequence data using the DADA2 pipeline (*42*). Only ESVs that were detected in more than one mesocosm in the conditioning stage [based on a larger dataset including 88 mesocosms and 26 plant species (*25*)] were included in data analysis (i.e., only sequences that were detected in two independent soil samples were analyzed). Microbial data were rarefied to the minimum sequence number per soil sample (13,023, 3833, and 3688 for fungal, bacterial, and protist data, respectively). Fungal sequencing data were split into three functional groups—putative pathogens, AM fungi, and saprotrophs—based on taxonomy [determined using the RDP classifier (*43*) and data available in FUNGuild (*26*)]. Sequences that had multiple function assignments in FUNGuild were excluded from the analysis. Seventy-seven ESVs of putative pathogens were recorded (41 were identified to species level comprising 18 species, and 36 were identified to genus level comprising 19 genera; the most common species were *Ilyonectria anthuriicola*, *Neonectria radicicola*, *Nectria ramulariae*, and *Olpidium brassicae*). AM fungi were represented with 126 ESVs (12, 42, 47, 18, and 7 were identified to species, genus, family, order, and class level, respectively, comprising 5 species, 8 genera, 5 families, 3 orders, and 1 class, respectively; the most abundant families were Paraglomeraceae, Glomeraceae, and Acaulosporaceae). Saprotrophs were represented by 338 ESVs (155 and 183 were identified to species and genus level, respectively; the most abundant genera were *Mortierella* and *Clavaria*). Detailed functional data are not currently available for soil bacteria and protists. We therefore only assessed the composition of bacterial and protist communities, and the relative abundance and richness of well-known pathogenic oomycete ESVs (*Pythium* and *Phytophthora* genera), as predictors of plant-soil feedbacks. We found that the composition of bacterial and protist communities was strongly correlated with that of saprotrophic fungi (table S2). Only the latter was included in the subsequent path analysis as a representative characteristic of overall microbial community composition.

Sequences were categorized as specialist if a particular sequence was detected in soil samples conditioned by fewer than 5 species and generalist if occurring in soil of more than 10 plant species (of 14 species in total). For each soil sample, the relative abundance, ESV richness, and exponential Shannon diversity (effective species number) were calculated for each fungal functional group and specialist and generalist sequences within each functional group. Asymptotic exponential Shannon diversity was calculated for each fungal group using the package iNEXT in R (*44*) to ensure that higher diversity values were not due to higher sequence read numbers belonging to a particular fungal group in some of the soil samples. However, very similar results were obtained using raw and asymptotic diversity estimates. In addition, to test whether plant-soil feedback was related to the abundance of a host-specific pathogen, indicator analysis of fungal pathogenic sequences was performed using the package indicspecies in R (*45*), and the relative abundance of the most host-specific pathogenic sequence was calculated for soil samples conditioned by each plant species. Calculated microbial abundance and diversity estimates were log_e_ transformed if necessary to satisfy the assumption of normal distribution before use in the models described below.

### Phylogenetic signal

The significance of phylogenetic signal in explaining the effects of plant species identity on soil microbial properties and plant-soil feedback was assessed using plant phylogeny from (*46*). The significance of Blomberg’s *K* and Pagel’s λ was estimated with randomization and likelihood ratio tests in the package phylosignal in R (*47*). In addition, Mantel tests were used to test whether phylogenetic distance between plant species explained the proportion of fungal pathogens, AM fungi, and saprotrophs that were distinct among species [assessed using Jaccard dissimilarity index based on presence-absence data; package vegan, function vegdist in R 3.4.0 (*48*)]. Given the limited number of plant species examined in this study, the power of the tests to detect phylogenetic signal was likely low.

### Multivariate data analysis and variance partitioning

Principal components analysis was performed on plant trait and soil abiotic properties data; the community composition of each fungal functional group (putative fungal pathogens, AM fungi, and saprotrophs) was characterized using principal coordinate analysis (using Bray-Curtis distances and Hellinger transformation of read abundance data). The first two principal coordinates for each fungal group and the first two principal component axes of plant traits and soil abiotic properties were used as predictors of plant-soil feedback in the analysis described below. The importance of plant species identity and the first principal component of abiotic soil properties in explaining variation in plant traits and soil microbial properties was analyzed using variance partitioning for univariate traits (comparison of linear fixed-effects models) and redundancy analysis for multivariate data [package vegan, function dbrda in R 3.4.0 (*48*)].

### Model selection and path analysis

The best predictors of soil feedback were selected on the basis of Akaike information criterion (AIC) from five groups of variables: plant traits, soil abiotic properties, and soil fungal community properties (pathogens, AM fungi, and saprotrophs). Each variable was tested in a separate linear model as a predictor of soil feedback, and predictors yielding the lowest AIC scores within each variable set were retained for use in the path analysis to explore possible paths by which plant traits and soil properties jointly affect soil feedback (table S1). Additional variables explaining more than 10% of variation in the feedback index, and not strongly correlated with the best predictor within each variable group, were also included in the path analysis. Within the set of abiotic soil properties, many variables were highly correlated and aligned closely with the first principal component. Hence, the latter was included in the path analysis to reflect the major axis of variation in abiotic soil properties. For the analysis of specific feedback, AIC scores with a sample size of 55 were calculated, as our dataset contained 55 mesocosms in the conditioning stage, representing true replicates in plant trait and soil measurements.

Path analysis was used to test whether fungal communities (putative pathogens, AM fungi, and saprotrophs) in conspecific and heterospecific soil affected plant-soil feedback and whether soil abiotic properties and plant traits have a direct or microbially mediated indirect effect on plant-soil feedback. Plant traits could be affected by soil abiotic conditions [i.e., phenotypic plasticity (*20*)]. In addition to direct effects of traits and abiotic conditions on saprotroph abundance and diversity, saprotrophs could also be affected by pathogens and AM fungi via changes in the rate of tissue death and litter properties as well as direct biotic interactions between different fungal guilds (*32*–*34*). Paths with *P* values higher than 0.1 were sequentially dropped (table S4).

For specific plant-soil feedback, the effects of plant traits and soil abiotic and biotic properties were explored separately for soils conditioned by conspecifics and heterospecifics (table S5). For conspecific soil properties, paths significant at *P* < 0.1 were retained. For heterospecific soil properties, no significant paths were identified and paths with a significance of *P* < 0.4 were retained to illustrate the contrast in the strength of paths in conspecific versus heterospecific soils. In the final model, all retained properties from the conspecific and heterospecific soil models were combined into a single path analysis.

### Dissimilarity in soil microbial communities and plant-soil feedback

To confirm that the diversity of putative pathogens and AM fungi were better predictors of biotic feedback than differences in fungal composition, Mantel tests were performed to assess the correlation between biotic feedback and fungal composition (based on Bray-Curtis distances and Hellinger-transformed data). The strength of this correlation was compared to the correlation between biotic feedback and fungal diversity obtained from the analogous Mantel test.

To test for the sensitivity of specific soil feedback to variation in soil fungal composition, pairwise dissimilarities between individuals of each species grown in conspecific versus heterospecific soils were calculated using log-transformed biomass and fungal sequencing data. Microbial dissimilarity was calculated as the Bray-Curtis distance based on the relative abundance data with Hellinger transformation [package vegan, function vegdist in R 3.4.0 (*48*)]. Separate distance matrices were constructed for three functional guilds: putative pathogens, AM fungi, and saprotrophs. The significance of a positive relationship between biomass distance and microbial community distance was tested for each plant species using 999 restricted permutations (within conspecific and heterospecific treatments within each plant species).

## Supplementary Material

http://advances.sciencemag.org/cgi/content/full/4/11/eaau4578/DC1

## SUPPLEMENTARY MATERIALS

Supplementary material for this article is available at http://advances.sciencemag.org/cgi/content/full/4/11/eaau4578/DC1 Data file S1. Data key. Data file S2. Plant trait data. Data file S3. Soil properties data. Data file S4. Feedback phase data. Fig. S1. Principal components analyses of soil abiotic properties and plant traits and variance partitioning analysis of plant traits and soil microbial community composition. Fig. S2. Heatmap of putative fungal pathogen sequences detected in each soil sample at the end of the soil conditioning phase. Fig. S3. Heatmap of AM fungal sequences detected in each soil sample at the end of the soil conditioning phase. Fig. S4. Plant dry mass at the end of the conditioning stage and the feedback stage when grown on live or sterilized conspecific soil or soil previously occupied by other species. Fig. S5. The contribution of soil abiotic properties, characteristics of soil fungal communities, and plant traits, to explaining variation in plant-soil feedbacks. Fig. S6. The proportion of shared putative pathogenic, AM and saprotroph fungi as a function of the phylogenetic distance between plant species and the frequency distribution of pathogenic, AM and saprotroph fungi in relation to the number of host plant species. Table S1. Selection of the best predictors of biotic and specific plant-soil feedbacks. Table S2. Relationships between soil bacterial and protist community composition and fungal saprotroph community composition, soil abiotic properties, and plant-soil feedback strength. Table S3. The relationship between the relative biomass difference between plants grown on conspecific versus heterospecific soils and the dissimilarity in fungal pathogenic, AM, and saprotroph fungal communities between these soils. Table S4. Full path analysis model of biotic plant-soil feedback and model simplification by the removal of nonsignificant links. Table S5. Full path analysis model of specific plant-soil feedback and model simplification by the removal of nonsignificant links.
