# Supplementary material for: Fungal diversity regulates plant-soil feedbacks in temperate grassland
Source: Sci Adv. 2018 Nov 28;4(11):eaau4578. doi: 10.1126/sciadv.aau4578 (PMC6261650; doi:10.1126/sciadv.aau4578)
Supplement: http://advances.sciencemag.org/cgi/content/full/4/11/eaau4578/DC1 [file supp_4_11_eaau4578__index.html]

Science Advances | Science Advances

## Supplementary Materials

**The PDF file includes:**

- Fig. S1. Principal components analyses of soil abiotic properties and plant traits and variance partitioning analysis of plant traits and soil microbial community composition.
- Fig. S2. Heatmap of putative fungal pathogen sequences detected in each soil sample at the end of the soil conditioning phase.
- Fig. S3. Heatmap of AM fungal sequences detected in each soil sample at the end of the soil conditioning phase.
- Fig. S4. Plant dry mass at the end of the conditioning stage and the feedback stage when grown on live or sterilized conspecific soil or soil previously occupied by other species.
- Fig. S5. The contribution of soil abiotic properties, characteristics of soil fungal communities, and plant traits, to explaining variation in plant-soil feedbacks.
- Fig. S6. The proportion of shared putative pathogenic, AM and saprotroph fungi as a function of the phylogenetic distance between plant species and the frequency distribution of pathogenic, AM and saprotroph fungi in relation to the number of host plant species.
- Table S1. Selection of the best predictors of biotic and specific plant-soil feedbacks.
- Table S2. Relationships between soil bacterial and protist community composition and fungal saprotroph community composition, soil abiotic properties, and plant-soil feedback strength.
- Table S3. The relationship between the relative biomass difference between plants grown on conspecific versus heterospecific soils and the dissimilarity in fungal pathogenic, AM, and saprotroph fungal communities between these soils.
- Table S4. Full path analysis model of biotic plant-soil feedback and model simplification by the removal of nonsignificant links.
- Table S5. Full path analysis model of specific plant-soil feedback and model simplification by the removal of nonsignificant links.

Download PDF

**Other Supplementary Material for this manuscript includes the following:**

- Data file S1 (.txt format). Data key.
- Data file S2 (.csv format). Plant trait data.
- Data file S3 (.csv format). Soil properties data.
- Data file S4 (.csv format). Feedback phase data.

**Files in this Data Supplement:**

- Adobe PDF - aau4578\_SM.pdf
