# Supplementary material for: Fungal diversity regulates plant-soil feedbacks in temperate grassland
Source: Sci Adv. 2018 Nov 28;4(11):eaau4578. doi: 10.1126/sciadv.aau4578 (PMC6261650; doi:10.1126/sciadv.aau4578)
Supplement: http://advances.sciencemag.org/cgi/content/full/4/11/eaau4578/DC1 [file aau4578_SM.pdf]

Science Advances
AAAS

advances.sciencemag.org/cgi/content/full/4/11/eaau4578/DC1

Supplementary Materials for

Fungal diversity regulates plant-soil feedbacks in temperate grassland

Marina Semchenko*, Jonathan W. Leff, Yudi M. Lozano, Sirgi Saar, John Davison, Anna Wilkinson, Benjamin G. Jackson, William J. Pritchard, Jonathan R. De Long, Simon Oakley, Kelly E. Mason, Nicholas J. Ostle, Elizabeth M. Baggs, David Johnson, Noah Fierer, Richard D. Bardgett

*Corresponding author. Email: marina.semchenko@manchester.ac.uk

Published 28 November 2018, *Sci. Adv.* **4**, eaau4578 (2018)
DOI: 10.1126/sciadv.aau4578

The PDF file includes:

Fig. S1. Principal components analyses of soil abiotic properties and plant traits and variance partitioning analysis of plant traits and soil microbial community composition.
Fig. S2. Heatmap of putative fungal pathogen sequences detected in each soil sample at the end of the soil conditioning phase.
Fig. S3. Heatmap of AM fungal sequences detected in each soil sample at the end of the soil conditioning phase.
Fig. S4. Plant dry mass at the end of the conditioning stage and the feedback stage when grown on live or sterilized conspecific soil or soil previously occupied by other species.
Fig. S5. The contribution of soil abiotic properties, characteristics of soil fungal communities, and plant traits, to explaining variation in plant-soil feedbacks.
Fig. S6. The proportion of shared putative pathogenic, AM and saprotroph fungi as a function of the phylogenetic distance between plant species and the frequency distribution of pathogenic, AM and saprotroph fungi in relation to the number of host plant species.
Table S1. Selection of the best predictors of biotic and specific plant-soil feedbacks.
Table S2. Relationships between soil bacterial and protist community composition and fungal saprotroph community composition, soil abiotic properties, and plant-soil feedback strength.
Table S3. The relationship between the relative biomass difference between plants grown on conspecific versus heterospecific soils and the dissimilarity in fungal pathogenic, AM, and saprotroph fungal communities between these soils.
Table S4. Full path analysis model of biotic plant-soil feedback and model simplification by the removal of nonsignificant links.
Table S5. Full path analysis model of specific plant-soil feedback and model simplification by the removal of nonsignificant links.

Other Supplementary Material for this manuscript includes the following:

(available at advances.sciencemag.org/cgi/content/full/4/11/eaau4578/DC1)

Data file S1 (.txt format). Data key.
Data file S2 (.csv format). Plant trait data.
Data file S3 (.csv format). Soil properties data.
Data file S4 (.csv format). Feedback phase data.

Supplementary materials

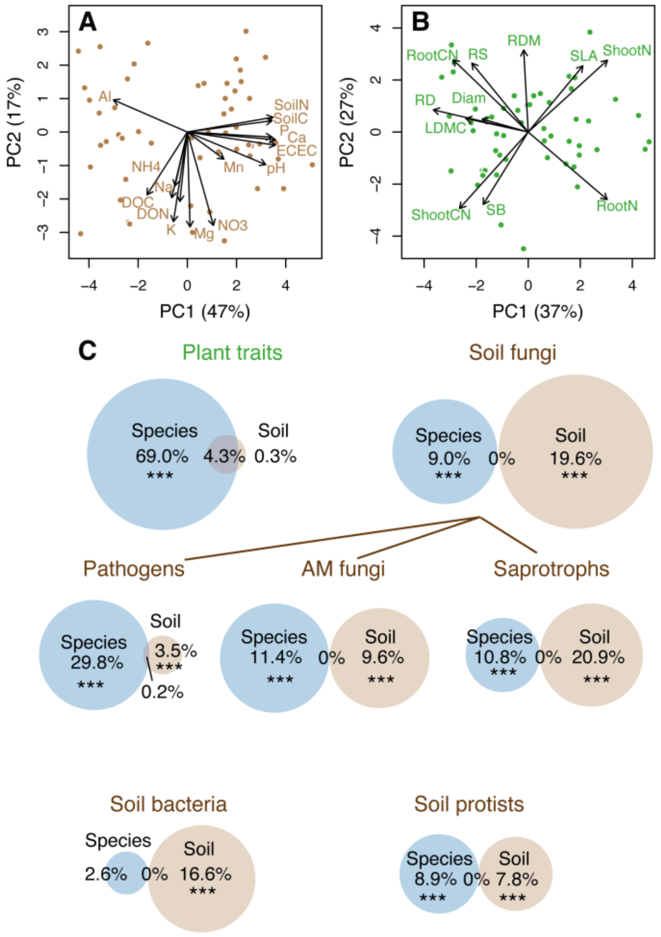

Fig. S1. Principal components analyses of soil abiotic properties and plant traits and variance partitioning analysis of plant traits and soil microbial community composition. Principal component analyses of abiotic soil properties (A) and plant traits (B), and variance partitioning analysis of plant traits and soil fungal, bacterial and protist community composition as a function of plant species identity and the first principal component of soil abiotic properties (C). Significance of factors in variance partitioning: * - $P < 0.05$; ** - $P < 0.01$; *** - $P < 0.001$ (n = 55). % variance explained is based on adjusted R^2. DOC – dissolved organic carbon; DON – dissolved organic N; ECEC – effective cation exchange capacity; RD - root dry mass density; Diam - root diameter; LDMC - leaf dry matter content; RDMC - root dry matter content; SLA - specific leaf area; SB - shoot biomass; RS - root to shoot biomass ratio; N – nitrogen % content; CN - carbon to nitrogen content ratio. Soil conditioning by different plant species did not significantly affect the first principal component of soil abiotic properties (adjusted R^2 = -0.06, P = 0.706), but plant species identity explained a significant proportion of variation in the second principal component (adjusted R^2 = 0.39, $P < 0.001$).

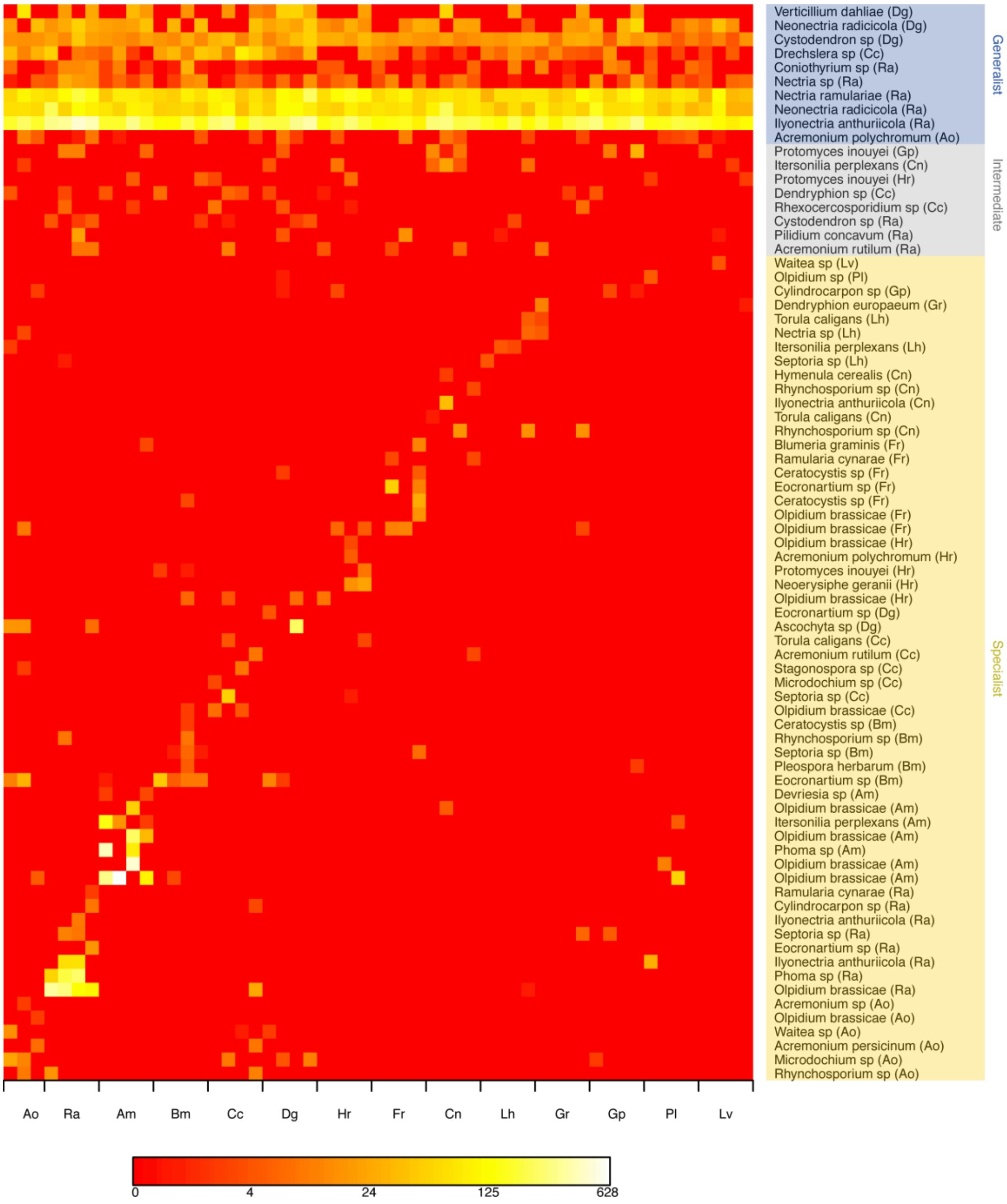

Fig. S2. Heatmap of putative fungal pathogen sequences detected in each soil sample at the end of the soil conditioning phase. The soil samples are grouped by plant species on the horizontal axis with species characterised by the highest number of specialist pathogenic exact sequence variants (ESVs) on the left (see Fig. 1 for plant species abbreviations and pathogen ESV richness). Putative pathogen ESVs and their most probable taxonomic identity are displayed on the vertical axis. ESVs are grouped into generalists, intermediate and specialists based on the number of host plant species that they occurred in

(generalists – more than 10 host species, specialists – fewer than five host species). ESVs are sorted by plant species that they colonised most preferentially based on the indicator species analysis. Plant species that showed the highest indicator value for a particular ESV is shown abbreviated in parentheses following fungal taxonomy.

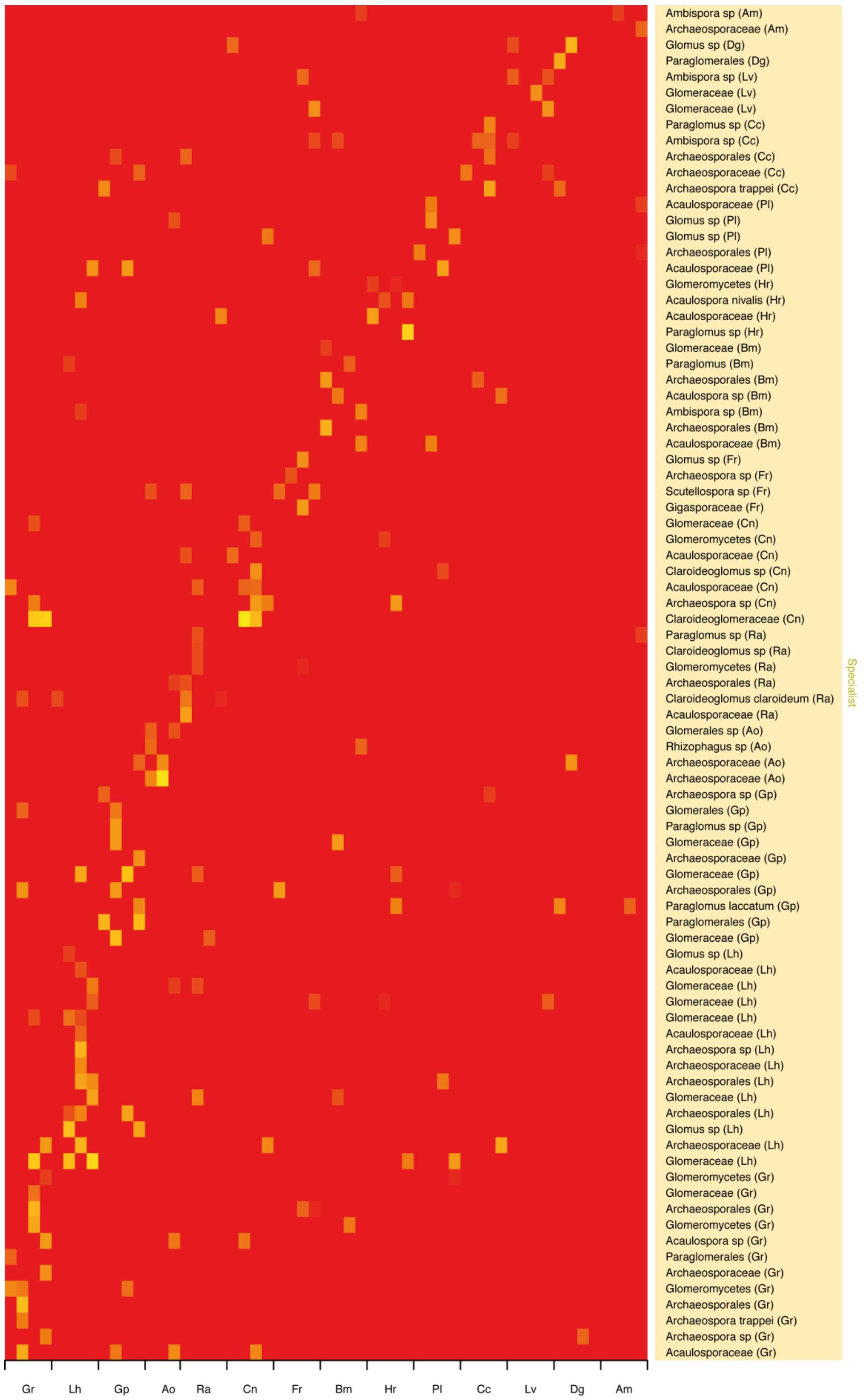
Ambispora sp (Am)
Archaeosporaceae (Am)
Glomus sp (Dg)
Paraglomerales (Dg)
Ambispora sp (Lv)
Glomeraceae (Lv)
Glomeraceae (Lv)
Paraglomus sp (Cc)
Ambispora sp (Cc)
Archaeosporales (Cc)
Archaeosporaceae (Cc)
Archaeospora trappei (Cc)
Acaulosporaceae (Pl)
Glomus sp (Pl)
Glomus sp (Pl)
Archaeosporales (Pl)
Acaulosporaceae (Pl)
Glomeromycetes (Hr)
Acaulospora nivalis (Hr)
Acaulosporaceae (Hr)
Paraglomus sp (Hr)
Glomeraceae (Bm)
Paraglomus (Bm)
Archaeosporales (Bm)
Acaulospora sp (Bm)
Ambispora sp (Bm)
Archaeosporales (Bm)
Acaulosporaceae (Bm)
Glomus sp (Fr)
Archaeospora sp (Fr)
Scutellospora sp (Fr)
Gigasporaceae (Fr)
Glomeraceae (Cn)
Glomeromycetes (Cn)
Acaulosporaceae (Cn)
Claroideoglomus sp (Cn)
Acaulosporaceae (Cn)
Archaeospora sp (Cn)
Claroideoglomeraceae (Cn)
Paraglomus sp (Ra)
Claroideoglomus sp (Ra)
Glomeromycetes (Ra)
Archaeosporales (Ra)
Claroideoglomus claroideum (Ra)
Acaulosporaceae (Ra)
Glomerales sp (Ao)
Rhizophagus sp (Ao)
Archaeosporaceae (Ao)
Archaeosporaceae (Ao)
Archaeospora sp (Gp)
Glomerales (Gp)
Paraglomus sp (Gp)
Glomeraceae (Gp)
Archaeosporaceae (Gp)
Glomeraceae (Gp)
Archaeosporales (Gp)
Paraglomus laccatum (Gp)
Paraglomerales (Gp)
Glomeraceae (Gp)
Glomus sp (Lh)
Acaulosporaceae (Lh)
Glomeraceae (Lh)
Glomeraceae (Lh)
Glomeraceae (Lh)
Acaulosporaceae (Lh)
Archaeospora sp (Lh)
Archaeosporaceae (Lh)
Archaeosporales (Lh)
Glomeraceae (Lh)
Archaeosporales (Lh)
Glomus sp (Lh)
Archaeosporaceae (Lh)
Glomeraceae (Lh)
Glomeromycetes (Gr)
Glomeraceae (Gr)
Archaeosporales (Gr)
Glomeromycetes (Gr)
Acaulospora sp (Gr)
Paraglomerales (Gr)
Archaeosporaceae (Gr)
Glomeromycetes (Gr)
Archaeosporales (Gr)
Archaeospora trappei (Gr)
Archaeospora sp (Gr)
Acaulosporaceae (Gr)
Specialist
Gr
Lh
Gp
Ao
Ra
Cn
Fr
Bm
Hr
Pl
Cc
Lv
Dg
Am

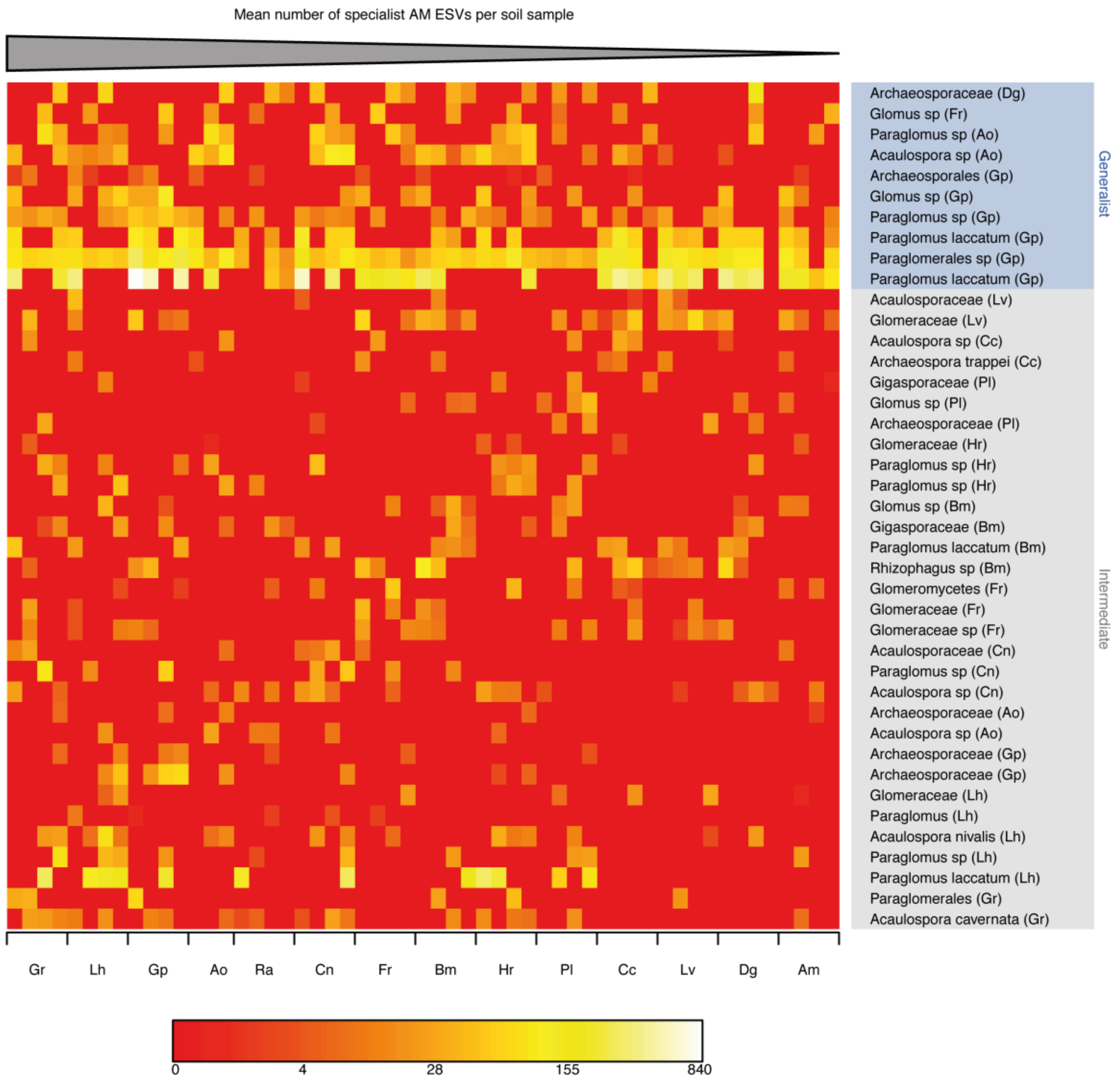

Fig. S3. Heatmap of AM fungal sequences detected in each soil sample at the end of the soil conditioning phase. The soil samples are grouped by plant species on the horizontal axis with species characterised by the highest number of specialist AM exact sequence variants (ESVs) on the left (see Fig. 1 for plant species abbreviations and AM fungal ESV richness). AM fungal ESVs and their most probable taxonomic identity are displayed on the vertical axis. ESVs are grouped into generalists, intermediate and specialists based on the number of host plant species that they occurred in (generalists – more than 10 host species, specialists – fewer than five host species). ESVs are sorted by plant species that they colonised most preferentially based on the indicator species analysis. Plant species that showed the highest indicator value for a particular ESV is shown abbreviated in parentheses following fungal taxonomy.

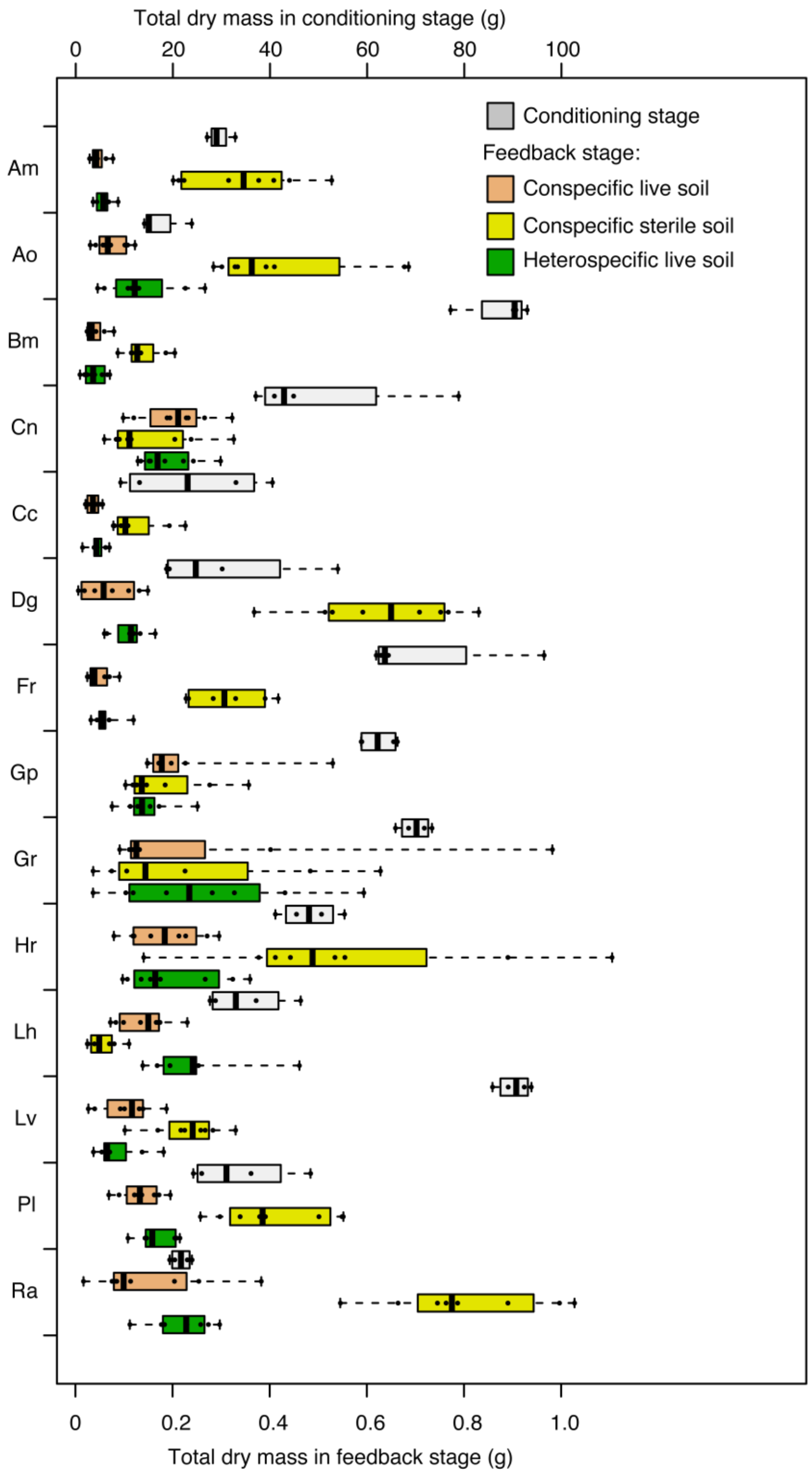

Fig. S4. Plant dry mass at the end of the conditioning stage and the feedback stage when grown on live or sterilized conspecific soil or soil previously occupied by other species. See Fig. 1 for species abbreviations. Medians (thick lines), 25 and 75 percentiles (box), and ranges (dashed lines) are shown.

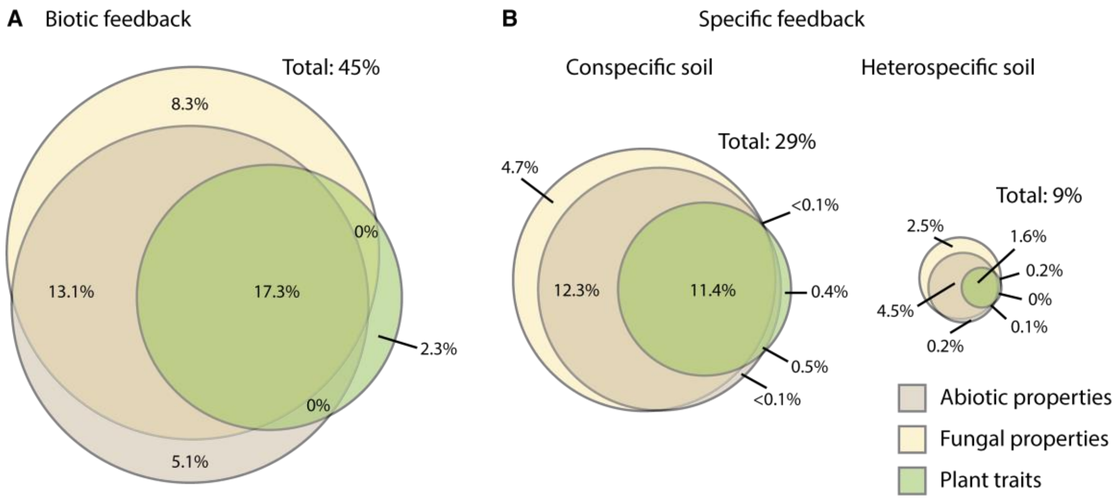

Fig. S5. The contribution of soil abiotic properties, characteristics of soil fungal communities, and plant traits, to explaining variation in plant-soil feedbacks. (A) Biotic feedback. (B) Specific feedback. Properties of soils occupied in the conditioning stage by the same (conspecific) and other plant species (heterospecific) are shown separately. Variance partitioning analysis included three sets of variables (abiotic, fungal and plant traits) that were selected as best predictors for further path analysis (highlighted in bold in table S1). % variance explained is based on adjusted R^2.

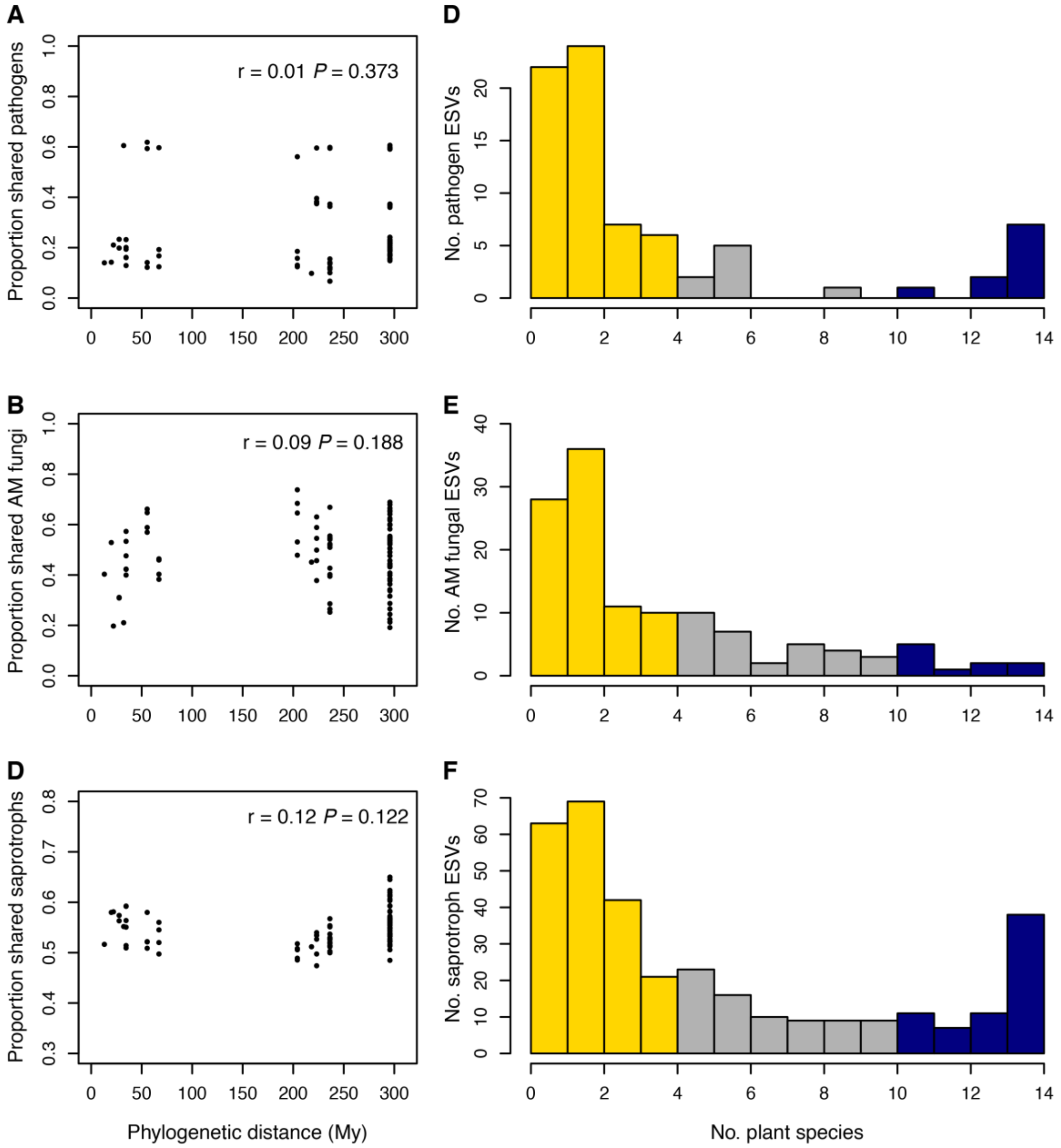

Fig. S6. The proportion of shared putative pathogenic, AM and saprotroph fungi as a function of the phylogenetic distance between plant species and the frequency distribution of pathogenic, AM and saprotroph fungi in relation to the number of host plant species. The dependence of the proportion of shared putative pathogens (A), arbuscular mycorrhizal (AM) fungi (B) and saprotrophs (C) on the phylogenetic distance between plant species; and the frequency distribution of pathogenic (D), arbuscular mycorrhizal (E) and saprotroph (F) fungal exact sequence variants (ESVs) in relation to the number of host plant species. Specialist sequences that occurred in the rhizosphere of fewer than five plant species are highlighted in yellow and generalist sequences occurring in more than ten host species are highlighted in blue; intermediate

sequences shown in grey. *P*-values and *r* are based on Mantel tests between phylogenetic distance and Jaccard dissimilarity matrices based on the presence-absence of sequences.

Table S1. Selection of the best predictors of biotic and specific plant-soil feedbacks. Properties of conspecific and heterospecific soils were tested in separate models (shown in separate columns). The fit of univariate linear models is shown with Akaike Information Criterion (AIC) and coefficient of determination (R^2). The number of observations is 55 for the biotic feedback and 818 for specific feedback (based on all pairwise combinations between plants grown on conspecific versus heterospecific soils). The variables selected for further path analysis are shown in bold.

	Biotic feedback		Specific feedback			
			Conspecific		Heterospecific	
**Plant traits**	AIC	R^2	AIC	R^2	AIC	R^2
1st principal component	175.3	0.07	**897.7**	**0.13**	904.7	0.01
2nd principal component	177.7	0.03	904.2	0.02	904.8	0.01
Shoot N%	**172.1**	**0.12**	899.0	0.11	905.2	<0.01
Root N%	178.4	0.01	902.8	0.04	904.8	0.01
$\log_e$(Leaf dry matter content,mg/g)	178.5	0.01	904.1	0.02	905.1	<0.01
$\log_e$(Root diameter, mm)	**170.3**	**0.15**	902.5	0.05	905.0	<0.01
$\log_e$(Root dry mass, g/cm^3 soil)	176.3	0.05	900.6	0.08	**904.1**	**0.02**
Shoot dry mass (g)	178.6	0.01	902.2	0.05	905.2	<0.01
Specific leaf area (m^2/kg)	177.1	0.04	900.4	0.08	905.2	<0.01
Root dry matter content, mg/g	175.0	0.07	904.5	0.01	905.0	<0.01
$\log_e$(Root:shoot biomass)	178.9	<0.01	905.0	<0.01	904.2	0.02
$\log_e$(Shoot C:N)	176.0	0.05	900.3	0.09	905.2	<0.01
$\log_e$(Root C:N)	178.3	0.01	903.0	0.04	904.8	0.01
**Abiotic soil properties**						
1st principal component	**174.9**	**0.07**	**893.4**	**0.19**	**901.5**	**0.06**
2nd principal component	171.4	0.13	903.9	0.02	905.1	<0.01
ECEC (cmol/kg)	176.3	0.05	894.2	0.18	901.2	0.07
pH	175.8	0.06	894.0	0.18	902.3	0.05
N%	176.6	0.04	898.4	0.12	901.7	0.06
C%	177.1	0.04	899.5	0.10	901.9	0.06
Total P (mg/kg)	175.4	0.06	896.6	0.15	902.5	0.05
$\log_e$(NO_3^-, mg N/kg)	**153.5**	**0.37**	**896.2**	**0.15**	904.8	0.01
$\log_e$(NH_4^+, mg N/kg)	178.8	<0.01	904.9	<0.01	905.1	<0.01
Dissolved organic N (mg/kg)	178.6	0.01	903.3	0.03	903.3	0.03
Dissolved organic C (mg/kg)	178.4	0.01	900.3	0.08	903.3	0.03
K (mg/kg)	179.1	<0.01	905.2	<0.01	904.8	0.01
log(Al, mg/kg)	175.5	0.06	893.7	0.19	902.0	0.06
Ca (mg/kg)	176.4	0.05	894.2	0.18	901.1	0.07
Mg (mg/kg)	167.3	0.19	903.4	0.03	905.2	<0.01
Mn (mg/kg)	177.3	0.03	902.0	0.06	905.1	<0.01
Na (mg/kg)	179.1	<0.01	905.0	<0.01	905.2	<0.01

Table S1 continued

	Biotic feedback		Specific feedback			
			Conspecific		Heterospecific	
**Soil fungi**	AIC	R^2	AIC	R^2	AIC	R^2
Putative pathogens						
1st principal coordinate	169.6	0.16	904.5	0.01	905.1	<0.01
2nd principal coordinate	178.8	0.00	903.3	0.03	905.2	<0.01
$\log_e$(Abundance)	171.2	0.13	902.9	0.04	905.2	<0.01
$\log_e$(Richness)	**162.3**	**0.26**	**897.6**	**0.13**	905.0	<0.01
$\log_e$(Diversity)	162.5	0.26	900.2	0.09	904.4	0.01
$\log_e$(Generalist abundance)	175.2	0.07	901.4	0.07	905.2	<0.01
Generalist richness	165.1	0.22	899.7	0.10	904.6	0.01
$\log_e$(Generalist diversity)	164.4	0.23	900.9	0.07	**903.8**	**0.02**
$\log_e$(Specialist abundance)	168.5	0.18	903.5	0.03	905.1	<0.01
$\log_e$(Specialist richness)	170.4	0.15	899.4	0.10	905.0	<0.01
$\log_e$(Specialist diversity)	172.8	0.11	899.2	0.10	905.1	<0.01
$\log_e$(Host-specialist abundance)	173.2	0.10	904.4	0.01	905.2	<0.01
Arbuscular mycorrhizal (AM) fungi						
1st principal coordinate	177.0	0.04	904.4	0.01	903.3	0.03
2nd principal coordinate	176.5	0.05	**901.0**	**0.07**	905.1	<0.01
$\log_e$(Abundance)	163.7	0.24	**901.7**	**0.06**	902.5	0.05
Richness	**163.3**	**0.25**	904.8	0.01	905.1	<0.01
Diversity	172.6	0.11	905.2	<0.01	905.1	<0.01
$\log_e$(Generalist abundance)	174.8	0.07	902.5	0.05	**901.9**	**0.06**
Generalist richness	170.0	0.15	904.0	0.02	904.9	<0.01
Generalist diversity	173.2	0.10	905.2	<0.01	905.2	<0.01
$\log_e$(Specialist abundance)	168.5	0.17	905.2	<0.01	905.2	<0.01
$\log_e$(Specialist richness)	170.8	0.14	905.2	<0.01	905.1	<0.01
$\log_e$(Specialist diversity)	171.8	0.12	905.1	<0.01	905.2	<0.01
Saprotrophs						
1st principal coordinate	176.1	0.05	**893.4**	**0.19**	**902.4**	**0.05**
2nd principal coordinate	178.7	0.01	904.8	0.01	905.0	<0.01
Abundance	178.9	<0.01	904.6	0.01	904.2	0.02
Richness	177.9	0.02	902.9	0.04	904.7	0.01
Diversity	178.9	<0.01	903.6	0.03	904.7	0.01
Generalist abundance	178.8	<0.01	904.5	0.01	903.9	0.02
Generalist richness	179.0	<0.01	905.0	<0.01	904.7	0.01
Generalist diversity	179.0	<0.01	904.7	0.01	904.5	0.01
$\log_e$(Specialist abundance)	179.1	<0.01	904.9	0.01	905.1	<0.01
Specialist richness	172.3	0.12	896.6	0.14	904.8	0.01
$\log_e$(Specialist diversity)	**168.1**	**0.18**	**896.1**	**0.15**	904.2	0.02

ECEC – effective cation exchange capacity; Host-specialist – fungal sequence with the maximum indicator value for a given plant species; Abundance – proportion of fungal sequences belonging to a particular microbial group; richness – the number of unique sequences (or exact sequence variants, ESVs); diversity – exponential Shannon diversity based on relative abundances of ESVs.

Table S2. Relationships between soil bacterial and protist community composition and fungal saprotroph community composition, soil abiotic properties, and plant-soil feedback strength. Pearson's correlations (r) and their statistical significance (*P*) are shown (based on n = 55). Composition of different microbial groups and soil fertility refer to the first principal coordinate of microbial composition and principal component of soil physicochemical properties. Pathogen oomycete abundance and richness refers to the relative abundance and richness of unique sequences belonging to *Pythium* and *Phytophthora* genera among protist sequences. Biotic feedback refers to plant growth response to the removal of soil biota by sterilisation and specific feedback refers to plant growth response to soil conditioned by conspecifics *versus* heterospecifics (more negative values mean greater growth on sterile soil or soil conditioned by other species, respectively). For specific feedback, the relationship with bacterial and protist composition in soil conditioned by conspecifics is shown.

	Bacteria composition		Protist composition		$\log_e$(pathogen oomycete abundance)		Pathogen oomycete ESV richness	
	r	*P*	r	*P*	r	*P*	r	*P*
Fungal saprotroph composition	0.97	<0.001	0.93	<0.001	-0.22	0.102	0.03	0.827
Soil fertility	0.91	<0.001	0.88	<0.001	-0.11	0.409	0.13	0.354
Biotic feedback	-0.22	0.101	-0.30	0.026	0.21	0.131	-0.12	0.402
Specific feedback	-0.43	<0.001	-0.49	<0.001	0.24	0.080	-0.06	0.678

Table S3. The relationship between the relative biomass difference between plants grown on conspecific versus heterospecific soils and the dissimilarity in fungal pathogenic, AM, and saprotroph fungal communities between these soils. Pearson's correlation coefficients (r) are shown for all data and each plant species separately. The significance (*P*) of a positive relationship between absolute differences in $\log_e$-transformed biomass and distances between soil communities were estimated using 999 restricted permutations (within conspecific and heterospecific treatment within each plant species and across all species, total n = 818). The last column shows the relationship between differences in biomass (absolute difference in $\log_e$-transformed biomass between each heterospecific soil and mean of conspecific soil) and phylogenetic distance between species that conditioned soil and species that occupied soil in the feedback stage of the experiment (total n = 104, *P*-values based on one-sided t-test). Correlations significant at *P* < 0.05 and 0.05 < *P* < 0.1 are shown in bold and italic, respectively.

Plant species	Pathogens		AM fungi		Saprotrophs		Plant phylogeny	
	r	*P*	r	*P*	r	*P*	r	*P*
All species	-0.06	0.793	0.03	0.291	**0.14**	**<0.001**	0.08	0.203
Achillea millefolium	-0.14	0.75	-0.13	0.767	0.16	0.199	0.36	0.212
Anthoxanthum odoratum	-0.05	0.551	0.02	0.46	**0.56**	**<0.001**	0.22	0.297
Briza media	0.06	0.321	-0.04	0.483	-0.25	0.895	0.08	0.430
Centaurea nigra	-0.20	0.943	0.07	0.289	0.07	0.271	-0.61	0.947
Cynosurus cristatus	-0.01	0.491	0.11	0.285	0.15	0.142	0.48	0.136
Dactylis glomerata	-0.15	0.848	0.10	0.431	0.06	0.384	-0.05	0.542
Festuca rubra	-0.06	0.563	0.10	0.343	-0.06	0.594	-0.55	0.899
Geranium pratense	0.24	0.167	*0.35*	*0.056*	0.22	0.16	0.47	0.145
Geum rivale	-0.05	0.601	*0.21*	*0.064*	**0.54**	**<0.001**	*0.59*	*0.063*
Hypochaeris radicata	0.10	0.234	0.14	0.109	**0.26**	**0.013**	-0.18	0.661
Leontodon hispidus	0.25	0.165	0.17	0.205	-0.08	0.648	0.25	0.291
Leucanthemum vulgare	<0.01	0.489	0.00	0.471	**0.30**	**0.013**	0.10	0.417
Plantago lanceolata	0.09	0.261	0.02	0.423	0.02	0.457	*0.64*	*0.061*
Rumex acetosa	-0.21	0.854	-0.36	0.932	-0.05	0.734	-0.44	0.862

Table S4. Full path analysis model of biotic plant-soil feedback and model simplification by the removal of nonsignificant links. Akaike Information Criterion (AIC) and Bayesian Information Criterion (BIC) of the full model and of each model after the removal of the least significant link, and the significance of χ^2 test comparing models with and without the least significant link (*P*), are shown.

Model	AIC	BIC	*P*
Feedback~AM_no+patho_no_log+sapro_spec_div_log+PC1_soil+ Diam_log+ShootN<br>AM_no~PC1_soil+Diam_log+ShootN<br>patho_no_log~PC1_soil+Diam_log+ShootN<br>sapro_spec_div_log~PC1_soil+Diam_log+ShootN+AM_no+ patho_no_log<br>Diam_log~PC1_soil<br>ShootN~PC1_soil<br>NO3_log~Diam_log+ShootN+con_fb+AM_no+patho_no_log+ sapro_spec_div_log<br>Diam_log~~ShootN	1058.5	1124.7	
NO3_log~patho_no_log	1056.5	1120.7	0.846
sapro_spec_div_log~ Diam_log	1054.6	1116.8	0.795
AM_no~Diam_log	1052.7	1112.9	0.759
NO3_log~ AM_no	1051.0	1109.3	0.533
sapro_spec_div_log~AM_no	1049.5	1105.7	0.497
Diam_log~PC1_soil	1048.0	1102.2	0.485
Feedback~ShootN	1047.1	1099.3	0.28
Feedback~PC1_soil	1046.2	1096.3	0.314
sapro_spec_div_log~PC1_soil	1045.5	1093.7	0.241
NO3_log~Diam_log	1045.4	1091.6	0.167
patho_no_log~Diam_log	1045.5	1089.6	0.155
AM_no~PC1_soil	1045.5	1087.7	0.154
Feedback~sapro_spec_div_log	1045.6	1085.8	0.144

n = 55; Feedback – biotic feedback, plant growth response to the removal of soil biota by soil sterilisation; AM_no – arbuscular myccorhizal fungal richness; patho_no_log – richness of putative plant fungal pathogens; sapro_spec_div_log – exponential Shannon diversity of specialist saprotrophic fungi; PC1_soil – first principal component of soil abiotic properties; Diam_log - root diameter; ShootN – shoot N%; NO3_log - soil nitrate concentration; log - $\log_e$ transformation.

Table S5. Full path analysis model of specific plant-soil feedback and model simplification by the removal of nonsignificant links. Akaike Information Criterion (AIC) and Bayesian Information Criterion (BIC) of the full model and of each model after the removal of the least significant link, and the significance of χ^2 test comparing models with and without the least significant link (*P*), are shown.

Model	AIC	BIC	*P*
Conspecific soil properties			
Feedback~AM_log+patho_no_log+PC2_AM+PC1_sapro+<br>sapro_spec_div_log+PC1_trait+PC1_soil+NO3_log<br>patho_no_log~PC1_trait+PC1_soil<br>AM_log~PC1_trait+PC1_soil<br>PC2_AM~PC1_trait+PC1_soil<br>PC1_sapro~PC1_trait+PC1_soil<br>sapro_spec_div_log~PC1_trait+PC1_soil+patho_no_log+AM_log+<br>PC2_AM<br>PC1_trait~PC1_soil<br>NO3_log~patho_no_log+AM_log+PC2_AM+PC1_sapro+<br>sapro_spec_div_log+PC1_trait	577.3	649.6	
sapro_spec_div_log~ PC1_soil	575.3	645.6	0.994
Feedback~PC1_soil	573.3	641.5	0.958
PC2_AM~PC1_trait	571.4	637.6	0.815
NO3_log~ PC1_sapro	569.4	633.6	0.818
Feedback~AM_log	567.5	629.7	0.811
Feedback~NO3_log	565.6	625.8	0.731
sapro_spec_div_log~ PC2_AM	563.7	621.9	0.737
NO3_log~ PC2_AM	561.9	618.1	0.660
Feedback~ PC2_AM	560.1	614.3	0.647
Feedback~PC1_trait	558.9	611.1	0.369
Feedback~patho_no_log	559.0	609.2	0.150
PC1_sapro~PC1_trait	559.2	607.4	0.134
Heterospecific soil properties			
Feedback~patho_gen_div_log+AM_gen_ra_log+PC1_sapro+<br>RD_log+PC1_soil<br>patho_gen_div_log~RD_log+PC1_soil<br>AM_gen_ra_log~RD_log+PC1_soil<br>PC1_sapro~RD_log+PC1_soil<br>RD_log~PC1_soil	325.4	359.5	
Feedback~PC1_sapro	323.4	355.5	0.987
Feedback~ RD_log	321.5	351.6	0.827
PC1_sapro~RD_log	319.5	347.6	0.802
patho_gen_div_log~RD_log	317.8	343.9	0.586

n = 55, Feedback – specific feedback, plant growth response to soil conditioned by conspecifics *versus* heterospecifics; AM_log – relative abundance of AM fungi; PC2_AM – second principal coordinate of AM fungal composition; patho_no_log – richness of putative plant fungal pathogens; PC1_sapro – first principal

coordinate of saprotroph composition; sapro_spec_div_log - Shannon diversity of specialist saprotrophic fungi; PC1_soil – first principal component of soil abiotic properties; PC1_trait - first principal component of plant traits; NO3_log - soil nitrate concentration; patho_gen_div_log - Shannon diversity of generalist plant pathogenic fungi; AM_gen_ra_log – relative abundance of generalist AM fungi; RD_log – root dry mass per unit soil volume; log - $\log_e$ transformation.
